# Distinct characteristics of asymmetric magnetic reconnections: Observational results from the exhaust region at the dayside magnetopause

**DOI:** 10.1038/srep27592

**Published:** 2016-06-08

**Authors:** Y. C. Zhang

**Affiliations:** 1 State Key Laboratory of Space Weather, National Space Science Center, Chinese Academy of Sciences, Beijing, 100190, China; 2 Key Laboratory of Earth and Planetary Physics, Institute of Geology and Geophysics, Chinese Academy of Sciences, Beijing, 100029, China

## Abstract

Magnetic reconnection plays a key role in the conversion of magnetic energy into the thermal and kinetic energy of plasma. On either side of the diffusion region in space plasma, the conditions for the occurrence of reconnections are usually not symmetric. Previous theoretical studies have predicted that reconnections under asymmetric conditions will bear different features compared with those of symmetric reconnections, and numerical simulations have verified these distinct features. However, to date, the features of asymmetric reconnections have not been thoroughly investigated using *in situ* observations; thus, some results from theoretical studies and simulations have not been tested with observations sufficiently well. Here, spacecraft observations are used in a statistical investigation of asymmetric magnetic reconnection exhaust at the dayside magnetopause. The resulting observational features are consistent with the theoretical predictions. The results presented here advance our understanding of the development of reconnections under asymmetric conditions.

Magnetic reconnections explosively convert magnetic energy into plasma kinetic energy and thermal energy over a wide range of plasma conditions, such as those observed in space, astrophysics and laboratory experiments^1^. The general definition of magnetic reconnection^2^ indicates that when different directed magnetic fields from either side of a diffusion region converge, they are broken and then reconnect with each other at X point. In nature, the plasma on either side of the diffusion region is generally not symmetric, and the reconnection process will develop on an asymmetric background^3^. For example, for typical conditions at the dayside magnetopause, the plasma on the magnetosheath side of the diffusion region has a low magnetic field, low temperature and high density, whereas the plasma on the magnetosphere side has a high magnetic field, high temperature and low density (Fig. 1).

One significant feature of asymmetric reconnections from theoretical analyses is that the X point (marked as X in Fig. 1) and the stagnant point (marked as S in Fig. 1) are not collocated as in symmetric reconnections^4^,^5^. The stagnant point will tilt towards the side with a smaller *ρ*/*B*, and the X point will tilt towards the side with a higher *β*, resulting in a net flow crossing the X point, as shown in Fig. 1. In the asymmetric reconnection layer, on the smaller *ρ*/*B* side, a slow expansion fan will change the plasma parameters, whereas on the high *β* side, a rotational discontinuity changes the direction of the magnetic field^6^.

Based on theoretical considerations and simulations, Cassak and Shay indicated that the reconnection scale depends on the asymmetric conditions on both sides of the diffusion region in an antiparallel reconnection^4^,^7^. For incompressible plasma, the scales of the out-flow speed (*v*_*out*_), out-flow density (*ρ*_*out*_), reconnection rate (*E*) and separating distance (*δ*_*XS*_) between the X point and the stagnant point are determined as follows, according to a Sweet-Parker type analysis^4^:


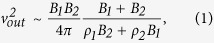



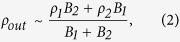



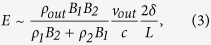



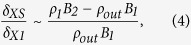


where subscripts 1 and 2 refer to the plasma conditions on either side of the diffusion region, *ρ* and *B* are the plasma density and reconnecting magnetic field, respectively, *δ*_*X1*_ is the distance from the X point to the border of the diffusion region, and *δ* and *L* are the width and length of the diffusion region, respectively, as shown in Fig. 1. Recently, the formula of the reconnection rate in equation (3) was tested using *in situ* observations^8^,^9^. However, to date, the other 3 scaling laws in the above equations have not been tested using *in situ* observations.

Although a number of studies have been performed on asymmetric reconnections based on theoretical principles and numerical simulations^4^,^5^,^7^,^10^,^11^,^12^, the features of reconnection under asymmetric conditions have not been verified clearly by observations. Because the dayside magnetopause usually provides an asymmetric plasma environment, reconnection events at this location are ideal candidates for the investigation of how the asymmetry across the diffusion region affects the occurrence of reconnections. Diffusion regions are rarely detected because of their small spatial scale; however, the exhaust regions adjacent to the diffusion region in the out-flow direction are much more likely to be observed. Previous studies have presented significant results for dayside reconnections based on analyses of the exhaust region^13^,^14^,^15^. In this report, we investigated the complete crossings of the reconnection exhaust at the dayside magnetopause based on the Double Star TC-1 satellite^16^ observations. Complete crossing indicates that each crossing consists of the following three parts: the exhaust region, the adjacent magnetosheath region and the magnetosphere region. We used the 4-s averaged magnetic field data from the Flux Gate Magnetometer (FGM)^17^ and ion data from the Hot Ion Analyser (HIA)^18^ on board the TC-1. We then statistically surveyed the asymmetric reconnection to determine how the reconnection develops under asymmetric conditions and compared the observational features with previous theoretical works and simulation results to determine whether these previous results are supported by the observations.

## Case study

**Figure 1:**
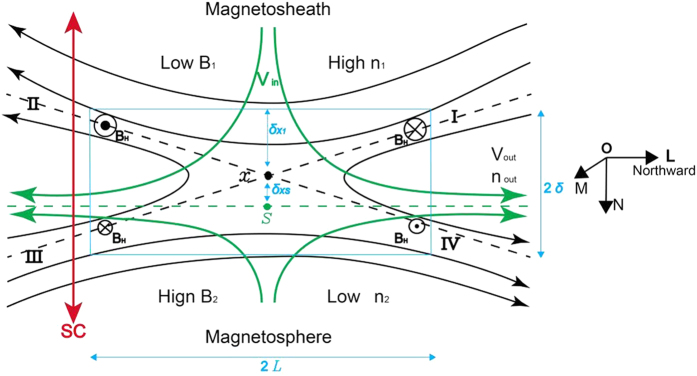
Sketch depicting the asymmetric antiparallel reconnection with the local magnetopause coordinates [L, M, N] superimposed at the right. The magnetosheath is at the top, and the magnetosphere is at the bottom. Solid black curves indicate magnetic field lines, and solid green curves indicate stream lines. Dashed black lines indicate the four separatrix lines, which intersect at the X point. Stagnant point S is located at the dashed green line, on which the flow velocity in the N direction is zero. The blue box marks the diffusion region with a width of 2*δ* and a length of 2*L*. The red line with the arrows at both ends indicates that the spacecraft crosses the exhaust region from the magnetosheath to the magnetosphere or vice versa.

**Figure 2:**
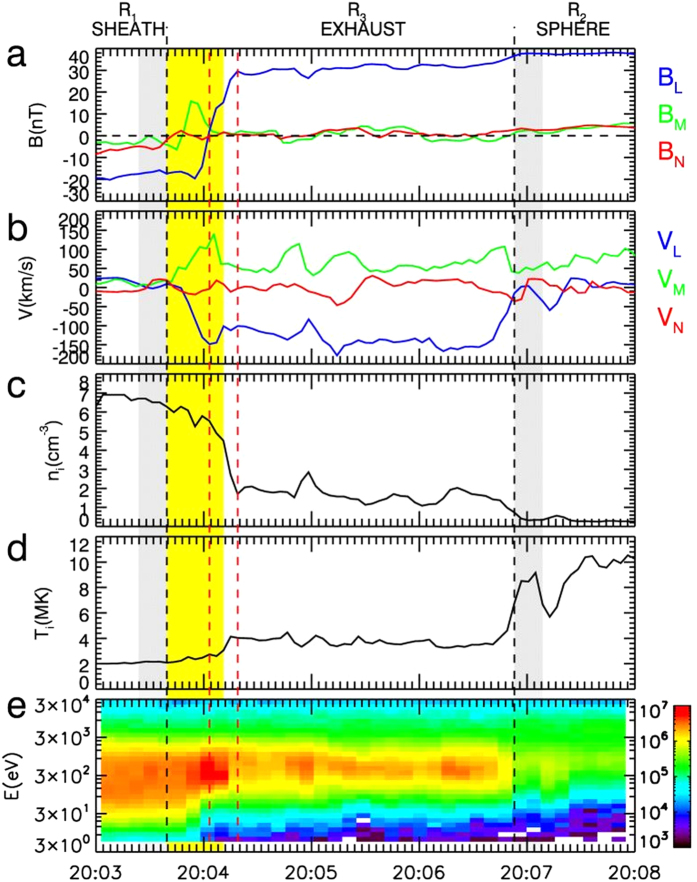
TC-1 observations of the asymmetric antiparallel reconnection event on May 15, 2006 in local LMN coordinates. **(a)** Magnetic field components; **(b)** ion bulk velocity components; **(c)** ion density; **(d)** ion temperature; and **(e)** ion energy spectrogram.

An asymmetric reconnection study is presented in this section to demonstrate how the study analysis is performed in this report. Between 20:03 UT and 20:08 UT on May 15, 2006, the TC-1 was moving inbound at (12.44, −1.74, 0.68) R_E_ (Earth Radius) in GSE (Geocentric Solar Ecliptic) coordinates. Figure 2 displays the observations of the TC-1 for that time interval in local LMN coordinates^19^ at the magnetopause. In our analysis, in local LMN coordinates at the dayside magnetopause, the axis ***L*** and the axis ***M*** point are approximately northward and duskward, respectively, and the axis ***N*** represents the inward normal vector to the magnetopause, as displayed in Fig. 1. The observations indicate 3 distinct regions separated by two vertical black dashed lines. Region R1 corresponds to the magnetosheath, which has a southward magnetic field, low magnetic intensity, low temperature and high plasma density; Region R2 corresponds to the magnetosphere, which has a northward magnetic field, high magnetic intensity, high temperature and low plasma density; and Region R3 corresponds to the exhaust region, which presents reconnection jets and has a moderate plasma density and temperature. Inside this exhaust region, there are obvious mixtures of magnetosheath hot ions and magnetosphere cold ions (Fig. 2e). The reconnection jet is aligned to the −L (southward) and +M (duskward) directions; therefore, the TC-1 crosses the southern branch of the exhaust region, which in this case, is labelled by the red spacecraft trajectory in Fig. 1. The peak of the reconnection jet (*V*_*out−obs*_) reaches up to 198 km/s, and the associated ion density (*n*_*out−obs*_) is 5.41 cm^−3^. Inside the exhaust region, the southward to northward rotation of *B*_*L*_ occurs at 20:04:04 UT, as indicated by the first red vertical dashed line, whereas the plasma features (density and temperature) have a sharp change 16 seconds later, indicated by the second red vertical dashed line. It is interesting to note that the separation of the *B*_*L*_ rotation point (BRP) and the plasma jump point (PJP) is apparent in this case. We will discuss this interesting feature later in this paper. To evaluate the background conditions of the magnetosphere side and the magnetosheath side, the average of 5 consecutive stable data points (16 sec time intervals without great fluctuations between the data points) adjacent to the exhaust region on either side are considered to be representative of the sheath and magnetosphere plasma. The data intervals to be averaged are shaded grey in Fig. 2. Only the tangential components [L, M] are considered for the evaluation of the background plasma conditions because they are the real components to be reconnected in the reconnection process. The plasma conditions in this case can be summarized as follows: at the sheath side, **B**_**1**_ = [*B*_*1L*_*, B*_*1M*_] = [−17.41 ± 1.12, −2.54 ± 1.02] nT, **V**_1_ = [V_*1L*_*, V*_*1M*_] = [16.06 ± 3.51, 15.97 ± 5.45] km/s, n_1_ =  6.74 ± 0.43 cm^−3^, and T_1_ = 2.05 ± 0.12 MK; and at the sphere side, **B**_2_ = [*B*_*2L*_*, B*_*2M*_] = [37.12 ± 1.31, 2.23 ± 0.51] nT, **V**_2_ = [V_*2L*_*, V*_*2M*_] = [−13.54 ± 10.01, 52.25 ± 8.84] km/s, n_2_ =  0.38 ± 0.11 cm^−3^, and T_2_ = 7.89 ± 0.91 MK. Thus, the plasma conditions are highly asymmetric, with *B*_*2*_/*B*_*1*_ = 2.14 ± 0.12 and *n*_*1*_/*n*_*2*_ = 17.73 ± 1.11. The shear angle (*θ*°) of the magnetic field lines between the sheath side and the magnetosphere side is 174.8° ± 8.9°, as estimated by *θ*° = acos (**B**_**1**_ ⋅ **B**_**2**_). The reconnection, in this case, developed under highly asymmetric and nearly antiparallel conditions. The intensity of the shear flow (*V*_*shear*_, the difference between [V_*1L*_, *V*_*1M*_] and [V_*2L*_, *V*_*2M*_]) is 63.3 ± 10.3 km/s. If all of the ions are regarded as protons, then inserting *B*_1_, *B*_2_, *n*_1_ and *n*_2_ into equations (1)–(2), 2 produces the predicted scales of the out-flow speed (*v*_*out*_ = 247.45 ± 16.38 km/s) and out-flow density (*n*_*out*_ = 4.7 ± 0.57 cm^−3^) for this antiparallel reconnection event. It should be noted that it is not the total magnetic magnitude that is used; rather, only the reconnecting fields *B*_1_ and *B*_2_ are used in the calculation of the predicted out-flow speed and out-flow density. We evaluated the deviation of the observed out-flow speed (*V*_*out−obs*_) from the theoretic prediction (*V*_*out*_) using Δ*V* = (*V*_*out*_ − *V*_*out−obs*_)/*V*_*out*_ and then applied the same evaluation to the deviation of the out-flow density Δ*n*. In this case, the values of Δ*V* and Δ*n* are 19% and 15%, respectively; therefore, the observed out-flow speed and out-flow density do not deviate much from the predicted values. Note that the out-flow plasmas from the X points will experience a gradually accelerating process to achieve the final out-flow speed at some distance from the X points. However, the evaluation of the distance of the spacecraft to the X point is an unsolved problem in the present study of magnetic reconnection. Thus, in this study, the extent to which the observed reconnection jets can precisely represent the final outflow speed is unknown. Nevertheless, we can gain some insight into this problem by comparing the reconnection jets to the local Alfven speed. In theory, the out-flow speed has a comparable value to that of the local Alfven speed^20^. As shown by the Walen test results in Methods section later, in most cases of this study, the observed reconnection jets can match the local Alfven speed; thus, it is reasonable that we take the observed jets as the proxy of the out-flow speed when compared to the predicted value. The *B*_*M*_ distribution in the exhaust region displays strong asymmetry (Fig. 2a) and has a mountain-type distribution with a top value of 17 nT at the sheath side and a flat distribution with a value of 1.4 nT at the magnetosphere side. The value of *B*_*M*_ at the sheath side of the exhaust region has the same direction as the duskward-directed Hall field (region II in Fig. 1). In the case of an antiparallel reconnection, the *B*_*M*_ can approximately be the proxy of the Hall field (Fig. 1); thus, in this case, the Hall fields are on the sheath side of the exhaust region, but nearly disappear on the magnetosphere side.

## Results and Discussion

We surveyed the TC-1 satellite magnetopause crossings with jet flows between 10LT and 14LT over the four-year period from 2004 to 2007. The local time (LT) range is confined to 10LT–14LT. In this limited dayside area, the asymmetry across the magnetopause is stronger, and reconnection is affected by the shear flow to a lesser extent compared with that in the flank region^21^. These crossings are verified by following the strict procedure presented in the study by Phan *et al.*^15^ to determine whether jet flows are produced by reconnection. First, the correlation between the change in flow and the change in the magnetic field is verified according to the reconnection scenario, as displayed in Fig. 1. For consistent events, the Walen test is performed^20^, and if the results from the Walen test do not support the occurrence of reconnection, then the ion distribution at the magnetopause crossing is verified to determine whether there is an ion mixture caused by the inter-connected magnetosheath and magnetosphere fields^22^. Eventually, 78 cases were verified as reconnection events in the exhaust region crossings.

### Separation of BRP and PJP in the Exhaust Region

**Figure 3:**
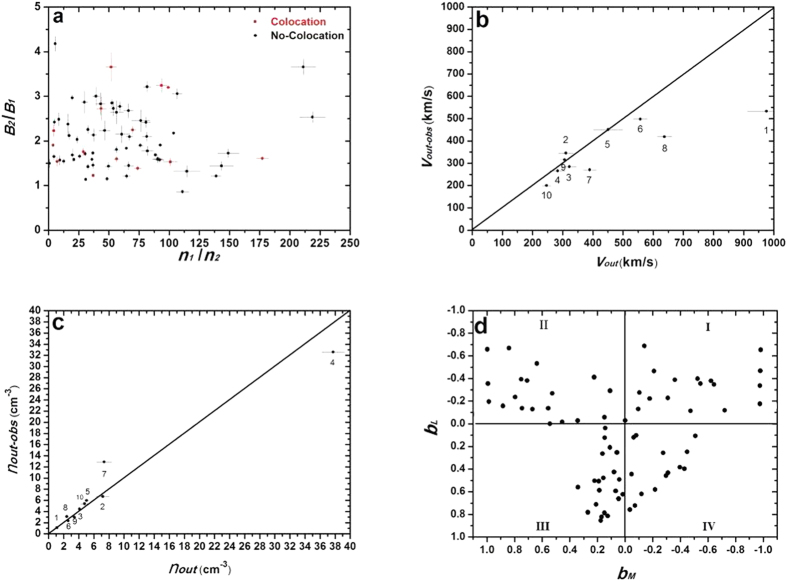
Statistical results for the asymmetric reconnection. **(a)** Asymmetric condition for the cases with non-collocation of the BRP and the PJP (black dots) and for the cases with collocation (red dots); **(b,c)** comparisons of the observed *V*_*out−obs*_ vs. the predicted *V*_*out*_ and the observed *n*_*out−obs*_ vs. the predicted *n*_*out*_, respectively; and **(d)** distribution of Hall fields in the exhaust region. The error bars in Fig. 3a–c indicate the standard deviation of the related parameters from the average of 5 separate data points, based on the definition of the background conditions in the magnetosphere and magnetosheath in section 2.

As shown in the above example, the non-collocation of BRP (marked by the first vertical red dashed line in Fig. 2) and PJP (marked by the second vertical red dashed line in Fig. 2) is apparently present in the asymmetric reconnection case. In Fig. 3a, all of the 78 exhaust region crossings are displayed in the asymmetric plasma conditions, where *B*_*2*_/*B*_*1*_ is the ratio of the magnetosphere field intensity to the sheath field intensity, and *n*_*1*_/*n*_*2*_ is the ratio of the sheath density to the magnetosphere density. In addition, 64 cases involving non-collocation are indicated by the black dots in Fig. 3a, and these cases cover a broad range of asymmetric conditions as follows: *1* < *B*_*2*_/*B*_*1*_ < 4 and *1* < *n*_*1*_/*n*_*2*_ < *255*. These conditions imply that the separation of the BRP and the PJP inside the exhaust region of asymmetric reconnection does not depend on the magnitude of the asymmetry. The rare 14 cases without separation (red dots in Fig. 3a) have similar asymmetric conditions to the cases with non-collocation, which also demonstrates that the magnitude of asymmetry is not a key factor in determining whether the separation of the BRP and the PJP occurs under asymmetric reconnection. The feature of the separation of the BRP and the PJP in the exhaust region has never been reported in previous references. However, we can obtain an explanation for this special feature from previous studies of diffusion region^4^. With the application of the conservation laws to the diffusion region, the separation between the X point and the stagnant point in the diffusion region has been theoretically predicted as a distinct feature of asymmetric reconnection^4^. In observations, it is very difficult to have the X point and the stagnant point meet at the centre of the reconnection diffusion region (Fig. 1). A realistic method is to use the BRP and the PJP as the proxies for the X point and the stagnant point inside the diffusion region, respectively^23^. In our statistical investigation of the separation of the BRP and the PJP in the exhaust region, we found the BRP is tilted towards the magnetosheath side and the PJP is tilted towards the magnetospheric side, in agreement with the features of the BRP and the PJP in the diffusion region^4^,^23^. Thus, we conclude that the separation of the BRP and the PJP in the exhaust region inherits this feature from the diffusion region. Because the investigation is performed at the exhaust region, the lack of observation of non-collocation of the BRP and the PJP in 14 cases may be caused by the evolution of the jet flow after its release from the diffusion region. In summary, the separation of the BRP and the PJP is a common feature of the exhaust region in asymmetric reconnections, regardless of the magnitude of the asymmetry. The exhaust region will generally move inward towards the magnetosphere as reconnection erodes the magnetopause^24^ because of the relative motion between the exhaust region and the TC-1; thus, the precise features of the separation distance as illustrated by *δ*_*xs*_ in equation 4 cannot be achieved based on single-point observations. This type of investigation will be the topic of the analysis of the data resulting from the anticipated Magnetospheric Multiscale mission (MMS)^25^.

### Cassak-Shay theory test

Cassak and Shay performed a theoretical analysis to deduce the asymmetric antiparallel reconnection scales in equations (1)–(4, 2, 3, 4) 4 and verified the scales using simulations^4^,^7^. Here, we verify their theoretical predictions of the out-flow speed (equation 1) and the out-flow density (equation 2) using *in situ* observations. Because a strictly antiparallel reconnection is rarely observed, we confine the criterion for the shear angle to more than 170° when selecting cases. A shear angle of 170° corresponds to an approximately 8% guiding field of the reconnected field. Simulations and laboratory experiments show that a guiding field of less than 10% does not produce noticeable changes in the reconnection features compared with the run without a guiding field^26^,^27^,^28^; therefore, a reconnection with a shear angle of more than 170° is nearly an antiparallel reconnection. Ten rare cases (listed in Table 1) satisfying the above criterion are selected from the 78 reconnection exhaust-region crossings. The same analysis described in the previous case study is performed on these 10 cases. The resulting features are listed in Table 1.

A clear demonstration of the plot of the observed out flow *V*_*out−obs*_ vs. the predicted out flow *V*_*out*_ is displayed in Fig. 3b, and the observed ion density *n*_*out−obs*_ vs. the predicted density *n*_*out*_ is displayed in Fig. 3c. In Fig. 3b,c, the dots associated with the numbers indicate the antiparallel cases in Table 1. The diagonal lines indicate where the observed values are equal to the predicted values. As shown in Fig. 3b, Cases 2 and 9 slightly deviate upward from the diagonal lines, indicating that the observed out flows in these cases are only slightly greater than the predicted value. The values of *V*_*out*_ and *V*_*out−obs*_ are nearly equivalent in Case 5; thus, this case is located on the diagonal line. The other 7 cases (Cases 1, 3, 4, 6, 7, 8, and 10) deviate downward from the diagonal line and have lower observed out flows than the predicted values. For 7 cases (Cases 2, 3, 4, 5, 6, 9, and 10), the Δ*V* values were less than 20%, whereas for cases 1, 7, and 8, the deviations were 45%, 30%, and 34%, respectively. The large deviations in these 3 cases may have been caused by the effects of the shear flow. Cassak demonstrated that shear flow can affect the tension release of newly reconnected field lines, which are responsible for the accelerating out flows^29^. Thus, the shear flow can play a role in reducing the speed of the out flow^5^. It is interesting to note that the shear flow in Cases 1, 7, and 8 is 175.41 km/s, 150.07 km/s and 126.40 km/s, respectively. These values rank in the top 3 of all shear flows in the 10 antiparallel cases; therefore, it is reasonable to attribute the large deviation of out flows in these three cases to the strong shear flows associated with the reconnection. Obvious shear flows are present in all 10 cases, which can also explain why most of the cases have reduced out-flow speeds compared with the predicted value. Doss *et al.*^5^ evaluated how the shear flow will affect the scales of asymmetric magnetic reconnection. Because that topic is beyond the scope of this study, the resulting scales in their study are not included in our out-flow calculation. In addition to the shear flow, the kinetic effects in reconnection may be another cause of the observed decreased out flow. The particle-in-cell (PIC) simulation on the asymmetric antiparallel reconnection by Malakit *et al.*^12^ demonstrated that the out-flow speed is consistent with the half of the predicted out-flow speed by MHD analysis^4^. Because of the limitation of observational ability, the kinetic effect on the out-flow speed is not investigated here. The observed ion density shows better consistency with the predicted values, as displayed in Fig. 3c. Case 7 has such a high observed density that the deviation value Δ*n* reaches up to 75%. The density deviations for the other 9 cases do not exceed 15%. In summary, for all the 10 investigated antiparallel reconnection cases, the observed out-flow speeds are strongly consistent with Cassak and Shay’s predictions for events with a weak shear flow. In addition, Cassak and Shay’s theory provides accurate predictions of the density in the out-flow region of asymmetric antiparallel reconnections.

### Hall magnetic field asymmetry

Collisionless reconnection is characterized by Hall fields in the diffusion region. However, the effect of asymmetry on Hall field distributions is an interesting topic that is not well understood. Because observations and modelling show that the presence of guiding fields significantly distort the topology of Hall fields^11^,^30^,^31^, precise investigations of the effects of asymmetric on Hall fields must exclude the effects of the guiding field; thus, only 10 antiparallel cases are analysed here. Because Hall magnetic fields are the result of Hall currents caused by the decoupling between ions and electrons in the collisionless reconnection diffusion region^32^, obvious Hall magnetic fields do not always occur in the reconnection exhaust region, as will be verified in our analysis. However, investigations of the Hall magnetic fields in the exhaust region of previous observations are still practical^33^,^34^,^35^. By verifying the M components of the magnetic fields representing the Hall magnetic fields of the antiparallel reconnections among the 10 antiparallel cases, 6 cases with obvious Hall magnetic fields are identified, as shown in Table 1. For the other 4 cases, the absence of Hall magnetic fields implies that the observed exhaust regions are located at a sufficient distance from the diffusion region so that the Hall magnetic fields cannot be observed.

Figure 3d displays the results of the Hall magnetic field distributions according to *b*_*L*_ vs. *b*_M_. In Fig. 3d, four quadrants labelled I, II, III and IV correspond to the regions labelled I, II, III and IV in Fig. 1, respectively. Each point indicates the normalized observed (*b*_*L*_, *b*_*M*_) values when the TC-1 crosses the exhaust regions that present a Hall magnetic field. The details of the normalization procedure are presented in the Methods section. As shown on the sheath side (−*b*_*L*_), the *b*_*M*_ distribution spreads over a wide range from −1.0 to 1.0, whereas on the magnetosphere side (+*b*_*L*_), the *b*_*M*_ distribution spreads over a narrow range from −0.5 to +0.4. The Hall magnetic field magnitudes are highly asymmetric and present a greater value on the sheath side, as indicated by B_H_ in Fig. 1. Using a PIC simulation on the asymmetric antiparallel reconnection, Malakit *et al.*^12^ found that the Hall magnetic field does not have a typical quadrupole structure but rather one bipolar structure on the high *β* side of the diffusion region (usually the magnetosheath side in observations). Although the total disappearance of Hall magnetic fields is not observed on the sphere side in all of the cases, as predicted by their simulations^12^, the statistical results of the weaker Hall magnetic fields on the sphere side are qualitatively consistent with their simulation results. A potential mechanism for Hall magnetic field asymmetry is related to the highly asymmetric distribution of the particle density on both sides of the diffusion region. Higher particle density on the sheath side can provide a greater carrier for the Hall current, resulting in more intense Hall currents and Hall magnetic fields. The particle asymmetry has been reported to play an important role in causing the asymmetric distribution of the Hall electric fields in the diffusion region^30^.

## Conclusions

In this study, we presented an analysis of the fast flow under asymmetric conditions based on TC-1 satellite observations of the dayside magnetopause (10LT–14LT) for the four-year period from 2004 to 2007. A total of 78 asymmetric reconnection exhausts were observed, and 10 rare antiparallel asymmetric reconnection cases were identified. Based on the statistical investigation of these cases, the features of asymmetric reconnections were deduced, and the following conclusions are presented.

Non-collocation is generally observed for the BRP and the PJP in the exhaust region of asymmetric reconnections. Whether non-collocation occurs is independent of the magnitude of asymmetry across the exhaust region. The exhaust region may inherit this special feature from the diffusion region, where the separation of the BRP and the PJP has been predicted and observed.

The results of the respective analysis of 10 rare antiparallel asymmetric reconnection cases with a shear angle of more than 170° provide observational evidence that supports the Cassak-Shay theory^4^. For the ion speed at the out-flow region, the predicted values are consistent with the observations for the 7 asymmetric antiparallel reconnection cases with a weak shear flow. For the ion density, the predicted and observed values are consistent for 9 of the 10 asymmetric antiparallel reconnection cases.

Hall magnetic fields occur in the exhaust region of 6 of the 10 antiparallel cases. Hall magnetic fields display strong asymmetry across the exhaust region and have a high magnitude on the sheath side. Because the guiding field effect is excluded in our antiparallel cases, this asymmetry should be related to the asymmetric condition, as shown in the simulations by Malakit *et al.*^12^.

A limitation of this study is that the exhaust regions are not the best locations to investigate the separation between X point and stagnant point and Hall field distribution. As shown in Fig. 1, the diffusion region is the ideal location for such an investigation; however, depending on the present observational ability, statistical results can only be achieved in the outer extension (the exhausts) of the inner diffusion region.

## Methods

### Walen test

The out flow from the magnetic reconnection is accelerated to the local Alfven speed ***V***_***A***_ = ***B***(*μ*_***0***_*ρ*)^***−1*****/*****2***^, where *ρ* is the mass density. By verifying whether the observed flow speed *V*′ can match the local Alfven speed, one can infer whether the flow is produced by magnetic reconnection^20^. ***V***′ = ***V*** − ***V***_***HT***_ is the flow speed observed in the de Hoffmann-Teller frame^36^, in which the electric field (***E***′) vanishes; in addition, Faraday’s law −(∂***B***/∂***t***) = ∇ × ***E*****′** = 0 indicates that the measured magnetic field structure is stationary in this frame.

**Figure 4:**
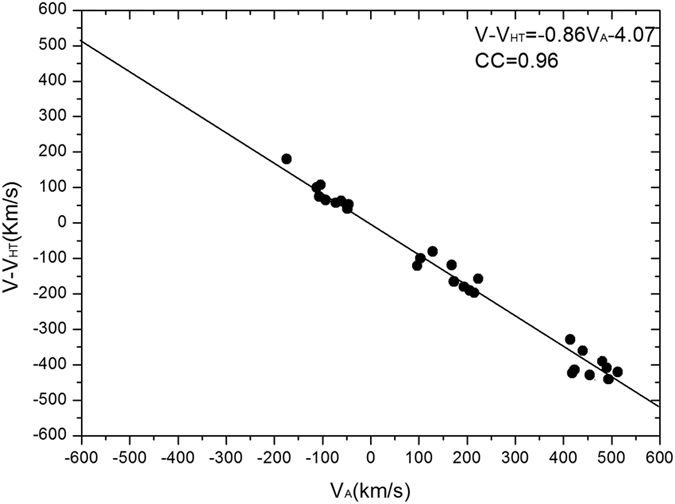
Results of the Walen test for the reconnection case on May 15, 2006. The data interval for the Walen test analysis is shaded in yellow in Fig. 2.

In Fig. 4, the results of the Walen test for our reconnection case study are displayed according to ***V***′ vs. ***V***_*A*_. The value of the correlation coefficient (CC) is used to evaluate the extent of the correlation between the calculated Alfven speed and the observed speed in the de Hoffmann-Teller frame. The high CC supports the occurrence of reconnection.

**Figure 5:**
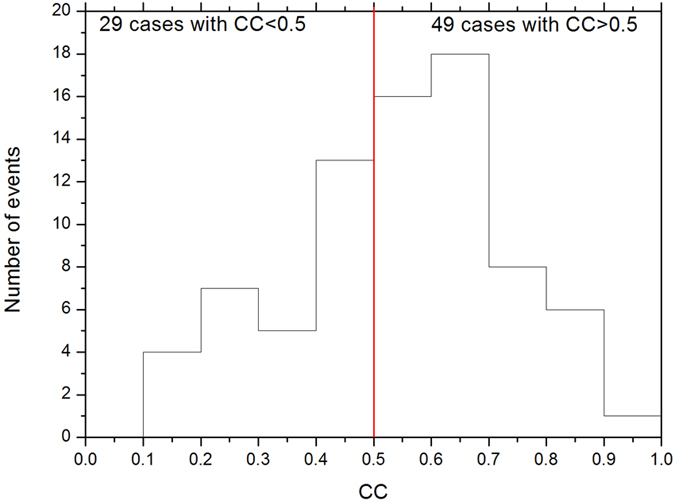
Distribution of the event numbers vs. the CC of the Walen test for all 78 reconnection cases.

Figure 5 presents the CC value distribution of the Walen test for all 78 reconnection cases in our study. The red vertical line marks the CC value of 0.5, and 49 cases with a CC > 0.5 are directly identified as reconnections. For the 29 cases with a CC < 0.5, the Walen test results are regarded as insufficient to support the occurrence of reconnection. Therefore, the ion distributions in these cases and the mixture of magnetosheath ions and magnetosphere ions are verified. The presence of the ion mixture implies that the magnetosphere and magnetosheath fields inside these jets are inter-linked because of magnetic reconnection. Thus, these cases are considered to represent the exhausts of reconnection.

### Normalization of Hall B fields

We use the normalized *b*_*L*_ with the value *b*_*L*_ = *B*_*LH*_/|*B*_*LB*_|. *B*_*LH*_ is the observed *B*_*L*_ at the Hall region, the value of which will gradually decrease to 0 from the boundary of Hall region to the centre of the Hall region. |*B*_*LB*_| is the magnitude of the L component of the referred background field adjacent to the Hall region. 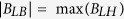 is at the magnetosphere side, and 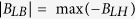 is at the magnetosphere side. As a result, the value of *b*_*L*_ can be observed as the relative distance of the position in the Hall region to the centre of the Hall region in every case.

The Hall B fields in the different cases have varied magnitudes. Thus, if we intend to display the distribution of different cases in one graph, then the distribution must be normalized within same physics regime. Although the laws of the Hall field distribution remain unclear, we gained insight that the Hall B fields are produced by Hall currents in the ion diffusion region. Thus, the fluctuating Hall fields in the same case have the same sources. In other words, the peak value of the Hall B field and the remaining values in the same case are intrinsically related by the same physical laws. We choose max(|*B*_*MH*_|) as the base for the normalization and normalize the value of *B*_*MH*_ to max(|*B*_*MH*_|), where *B*_*MH*_ are the observed Hall fields *B*_*M*_ at the Hall region, and max(|*B*_*MH*_|) is the maximum magnitude of *B*_*MH*_ for each case. The resulting value *b*_*M*_ = *B*_*MH*_/max(|*B*_*MH*_|) is used to show the distribution of the Hall B fields in different cases in the same picture and to see the asymmetric feature.

## Additional Information

**How to cite this article**: Zhang, Y. C. Distinct characteristics of asymmetric magnetic reconnections: Observational results from the exhaust region at the dayside magnetopause. *Sci. Rep.* **6**, 27592; doi: 10.1038/srep27592 (2016).

## Figures and Tables

**Table 1:** Features of the 10 asymmetric antiparallel reconnections^a^.

Case No.	Date	GSE Location (R_E_)	Shear Angle (degree)	n_out_ (cm^−3^)	n_out−obs_ (cm^−3^)	Δ*n*	V_out_ (km/s)	V_out−obs_ (km/s)	Δ*V*	V_shear_ (km/s)	Hall Field?
1	Feb 13, 2004	(9.54, 3.75, −1.68)	170.7 ± 12.7	1.09 ± 0.11	1.12	−3%	975.68 ± 63.99	533.25	45%	175.41 ± 20.51	Y
2	Apr 02, 2004	(9.93, −4.82, −1.31)	172.4 ± 7.9	7.18 ± 0.86	6.72	6%	310.62 ± 24.87	345.45	−11%	112.05 ± 14.67	Y
3	Apr 15, 2005	(12.20, −3.84, 3.02)	174.4 ± 14.2	4.08 ± 0.08	4.47	−9%	322.39 ± 23.92	284.75	12%	75.42 ± 24.56	Y
4	Apr 05, 2006	(7.63, −0.67, 3.11)	178.3 ± 11.2	37.77 ± 1.51	32.61	13%	283.41 ± 25.56	266.27	6%	72.41 ± 16,98	N
5	Apr 09, 2006	(10.15, 0.12, 3.29)	171.5 ± 10.5	5.04 ± 0.30	5.78	14%	450.28 ± 48.71	451.97	−0.003%	82.87 ± 23.67	N
6	Apr 17, 2006	(9.91, −1.22, 3.23)	172.6 ± 9.8	2.60 ± 0.21	2.33	10%	557.13 ± 23.89	497.66	11%	98.98 ± 19.87	Y
7	Apr 22, 2006	(8.12, 4.06, −0.48)	170.1 ± 11.7	7.36 ± 1.03	12.91	−75%	389.59 ± 22.76	270.98	30%	150.07 ± 30.56	Y
8	May 13, 2006	(9.96, −4.77, 2.95)	176.1 ± 10.8	2.80 ± 0.24	3.12	−11%	637.54 ± 25.13	418.96	34%	126.40 ± 19.45	N
9	May 15, 2006	(10.4, −5.00, 2.82)	170.4 ± 14.2	3.42 ± 0.28	3.01	12%	306.26 ± 15.17	316.37	−3%	80.01 ± 21.3	N
10	May 15, 2006	(12.44, −1.74, 0.68)	174.7 ± 8.9	4.70 ± 0.57	5.41	−15%	247.45 ± 16.38	198.77	19%	63.30 ± 10.3	Y

^a^Columns n_out_ and V_out_ indicate the predicted flow speed and the predicted plasma density in the out-flow region from equations (1)–(2), respectively. Columns n_out−obs_ and V_out−obs_ indicate the observed flow speed and the observed plasma density in the out-flow region from the TC-1, respectively. Column Hall Field indicates whether Hall fields are observed at the exhaust region.
